# Apigenin promotes neuronal norepinephrine release via GPR30/PLCβ1-Ca^2+^-Syt1 signaling to enhance astrocytic lactate and exert neuroprotection in D-galactose-induced cognitive impairment

**DOI:** 10.3389/fphar.2026.1979963

**Published:** 2026-10-26

**Authors:** Shan Jiang, Ling Kong, Xueke Bai, Meng Yang, Jiaxin Ding, Yaoai Wang, Wenjing Fan, Yan Yan, Chengyu Pu, Ning Zhang

**Affiliations:** State Key Laboratory of Integration and Innovation of Classic Formula and Modern Chinese Medicine, National Chinmedomics Research Center, National TCM Key Laboratory of Serum Pharmacochemistry, Metabolomics Laboratory, Department of Pharmaceutical Analysis, Heilongjiang University of Chinese Medicine, Harbin, China

**Keywords:** Alzheimer’s disease, apigenin, Ca^2+^ signaling, glycogen synthesis, lactate, neurons, norepinephrine

## Abstract

**Objective:**

AD, a prevalent neurodegenerative disorder clinically defined by progressive cognitive impairment, is pathologically featured by early dysfunction of the locus coeruleus, depletion of noradrenergic (NE) neurons, and dysregulated astrocytic glycogen metabolism, which collectively compromise patients’ daily functioning. Although Api, a common phytoestrogen, has shown therapeutic efficacy in diverse diseases, its role and mechanism in AD remain unexplored.

**Methods:**

To address this gap, we evaluated the protective effects of Api against D-gal-induced cognitive deficits through behavioral and histomorphological assessments. Untargeted metabolomics was performed to profile metabolic alterations in the locus coeruleus (LC), complemented by targeted metabolomics focusing on specific pathways. Putative targets were predicted by molecular docking and verified via cellular thermal shift assay (CETSA), while molecular biology approaches were adopted to elucidate the underlying signaling cascades.

**Results:**

Api significantly ameliorated D-gal-induced cognitive impairment and increased hippocampal NE and lactate levels with enhanced neuronal activity. Metabolomics indicated that Api regulates energy metabolism pathways including TCA cycle, amino acid metabolism, glycolysis/gluconeogenesis, and glutathione metabolism. Specifically, Api elevated NE and lactate in the LC. GPR30 was identified and validated as the key target via molecular docking and CETSA, showing strong binding affinity. Mechanistically, Api activates GPR30 on noradrenergic neurons to regulate PLCβ1-Ca^2+^-Syt1 signaling, promoting NE release, which in turn drives astrocytic glycogenolysis and lactate production, thereby relieving neuronal energy deficits.

**Conclusion:**

Collectively, this study not only clarifies the PLCβ1-Ca^2+^-Syt1-dependent mechanism of Api in aging-related cognitive impairment, but also highlights NE and lactate elevation as a promising therapeutic approach.

## Graphical Abstract

**Figure:**
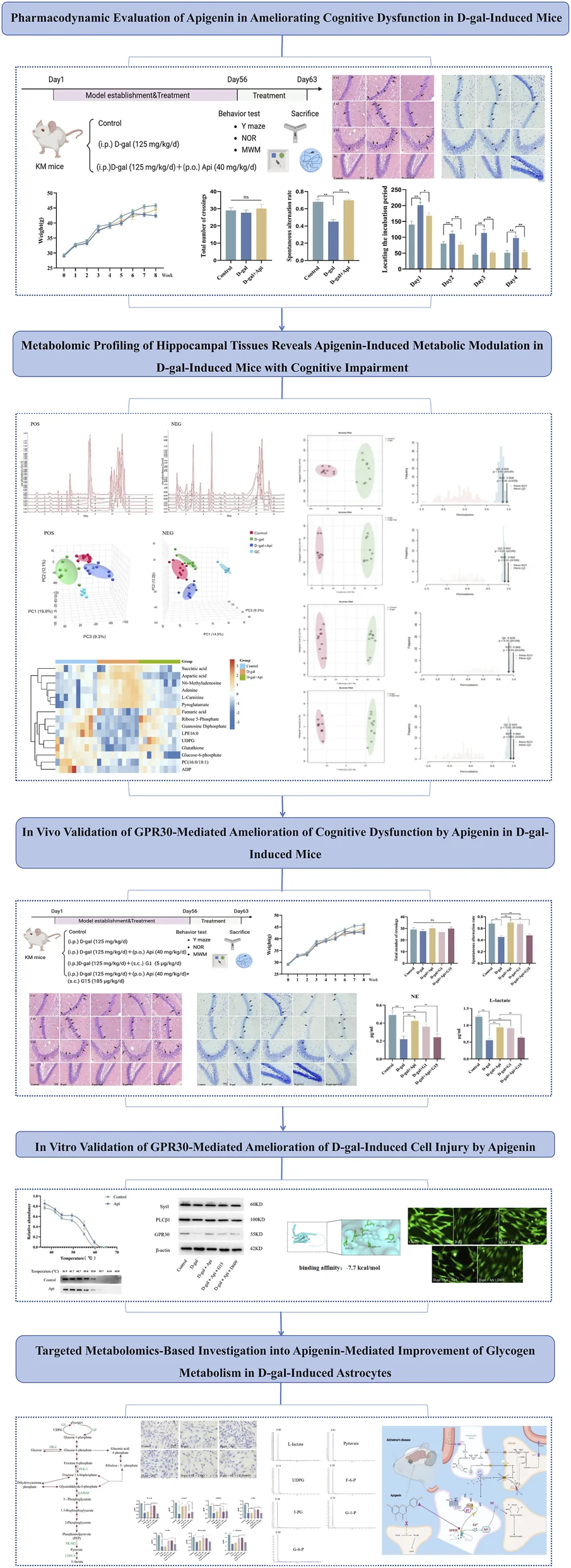

## Highlights

- Apigenin, derived from the classic formula Erzhi Wan, ameliorated cognitive deficits and restored hippocampal neuronal morphology in D-gal-induced AD model mice.
- GPR30 was identified and validated as a direct target of apigenin (molecular docking: −7.7 kcal/mol; CETSA).
- Apigenin promoted LC-NE release via the GPR30/PLCβ1-Ca^2+^-Syt1 signaling axis, a novel finding.
- A complete “NE → astrocytic glycogenolysis → lactate → ANLS → neuronal ATP” pathway was elucidated.
- Metabolomics revealed that apigenin modulates TCA cycle, amino acid metabolism, and glycolysis/gluconeogenesis.

## 1 Introduction

Alzheimer’s disease (AD) is a central neurodegenerative disorder commonly prevalent in the elderly population, characterized by cognitive decline, memory impairment, and progressive loss of daily living functions (Weller and Budson, 2018).

Glucose metabolism provides fuel for over 95% of ATP production in the brain, with neuronal ATP being primarily generated through the tricarboxylic acid (TCA) cycle and oxidative phosphorylation within mitochondria. Owing to its reliance on a single energy substrate, the brain is highly vulnerable to disturbances in energy metabolism (Magistretti and Allaman, 2018). Cerebral energy metabolic disturbances and persistent brain energy deficits lead to functional abnormalities in neurons and astrocytes, impair Aβ clearance and promote tau hyperphosphorylation in the brain, thereby triggering a vicious cycle of energy failure and functional deterioration. Therefore, preserving and restoring neuronal energy supply is of critical importance for preventing the onset and progression of Alzheimer’s disease (Cunnane et al., 2020).

Although the brain accounts for only 2% of total body mass, its energy demand is remarkably high, consuming approximately 20% of the body’s oxygen and 25% of its glucose exclusively for brain function (Bélanger et al., 2011). Among these processes, the maintenance and restoration of ion gradients dissipated by signal transduction events—such as postsynaptic and action potentials—along with neurotransmitter uptake and recycling, represent the primary contributors to the high energy demand of the brain (Onkar et al., 2023). Traditionally, neurons were thought to rely on a continuous supply of glucose. However, emerging evidence has revealed that brain glycogen dynamics play a critical role in neuronal energy homeostasis, particularly under energy stress and pathological conditions. Brain glycogen is predominantly stored in astrocytes and serves as a crucial energy reserve in the central nervous system (CNS) (Brown, 2004).

Astrocytes, through their perivascular endfeet, provide and process the physical interface between the vasculature and synaptic terminals, thereby supporting metabolic coupling between glial cells and neurons (Kacem et al., 1998). To reach neurons, the majority of glucose must first be transported through astrocytes (Rouach et al., 2008). Astrocytes are responsible for taking up over 60% of the brain’s glucose, yet they consume only 5%–15% of the brain’s energy demand. A small fraction of the glucose taken up by astrocytes is used for ATP production to sustain their own basal activities, while the majority is converted into glycogen for storage (López-Gambero et al., 2019). Under conditions of intense neuronal activity or metabolic stress—such as hypoglycemia or hypoxia—glycogen stored in astrocytes is continuously broken down to supply energy to neurons (Linnerbauer and Rothhammer, 2020).

The LC is a small nucleus located deep within the brainstem, providing the brain with an extensive noradrenergic neurotransmitter system. For over 50 years, the LC was considered structurally and functionally homogeneous, capable of projecting to a wide range of functionally diverse CNS regions. Consequently, its primary neurotransmitter, NE, was thought to be uniformly released and to act simultaneously across the entire forebrain, brainstem, cerebellum, and spinal cord. NE has been shown to exert neuroprotective effects against neurodegeneration and to play important roles in behavior and cognition (Poe et al., 2020). Within the hippocampus, the β_2_-adrenergic receptor (β_2_-AR) is the most abundantly expressed adrenergic receptor subtype. The LC-NE neurons project to various hippocampal subregions, forming the LC-hippocampal noradrenergic circuit (LC→HPC-NE), through which NE acts on hippocampal astrocytes via β_2_-AR (Giorgi et al., 2019). Studies have shown that NE modulates astrocytic uptake and release of glutamate and GABA, thereby regulating the excitatory/inhibitory balance of hippocampal neural networks (Park et al., 2021). These regulatory processes play critical roles in hippocampal-dependent learning and memory, particularly during the memory consolidation phase.

NE is primarily synthesized in noradrenergic neurons of the LC within the central nervous system. The essential precursor for NE noradrenergic neurons is tyrosine, which is converted to L-DOPA by tyrosine hydroxylase (TH), and subsequently to dopamine (DA) by DOPA decarboxylase (DDC). DA is then taken up into synaptic vesicles, where it is converted to NE by dopamine β-hydroxylase (DβH) and stored in the vesicles in association with ATP and chromogranins (Tao et al., 2024). During NE release, synaptic vesicles play a dual role: they determine the amount of NE released at nerve terminals and orchestrate the entire release process. When an action potential reaches the nerve terminal, the permeability of the presynaptic membrane changes, allowing Ca^2+^ influx, which promotes vesicle docking to the presynaptic membrane and membrane fusion, ultimately discharging the vesicular contents into the synaptic cleft. Calcium ions are critically involved in the regulation of vesicular exocytosis (Weinshilboum et al., 1971). Key proteins participating in neuronal and endocrine cell exocytosis include SNARE proteins, which constitute the core molecular machinery mediating vesicle fusion, and synaptotagmin 1 (Syt1), which serves as the Ca^2+^ sensor that triggers vesicle fusion. Each Syt1 protein can bind up to five Ca^2+^ ions and, through Ca^2+^-dependent interactions with membrane phospholipids and the SNARE complex, initiates the fusion of vesicles with the plasma membrane, thereby regulating neurosecretion (Papke et al., 2012).

Our previous studies have focused on the neuroprotective effects of Erzhi Wan (EZP), a traditional Chinese herbal formula, in the treatment of AD. We found that EZP ameliorated learning and memory deficits in D-galactose-induced aging mice and improved hippocampal neuronal morphology. These beneficial effects were attributed to multiple mechanisms, including the restoration of cerebral energy metabolism, attenuation of oxidative stress, and regulation of synaptic plasticity. Notably, apigenin is a common constituent of both Ligustri Lucidi Fructus and Ecliptae Herba, and is considered the primary bioactive component responsible for the therapeutic effects of EZP (Xue et al., 2025). Api is a flavonoid compound and a naturally occurring phytoestrogen. It is a major bioactive component of several medicinal plants, including Ecliptae Herba (Mohanlian), Plantaginis Semen (Cheqianzi), and Trachelospermi Caulis (Luoshiteng). A large body of *in vitro* and *in vivo* studies has demonstrated that phytoestrogens possess potential neuroprotective properties in the brain under various pathological conditions, ranging from aging to neurodegenerative diseases and cerebral ischemia, although further investigation is still warranted (Kujawski et al., 2026).

Estrogen receptors (ERs) are a class of proteins that specifically bind to estrogens, including nuclear receptors (ERα, ERβ) and the membrane-associated receptor (GPR30). ERα promotes the proliferation and differentiation of endometrial cells and regulates the menstrual cycle (Arnal et al., 2017). ERβ shares high homology with ERα, but differs in amino acid sequences in regions such as the LBD, resulting in distinct ligand-binding properties and biological effects (Saito and Cui, 2023). In the brain, ERβ is involved in neuronal growth, differentiation, and survival, and exerts significant effects on learning, memory, and cognitive function (Rymbai et al., 2024). Our previous studies also found that the active components of EZP, including apigenin and wedelolactone, promote the mRNA expression of TH and DβH via ERs, thereby enhancing NE synthesis and exerting anti-AD effects.

In the central nervous system, GPR30 is distributed in both neurons and glial cells and is involved in physiological processes such as neuroprotection and neurotransmitter regulation (Lin et al., 2026). One of the most important functions of GPR30 is its involvement in rapid cellular signaling, including PI3K activation, Ca^2+^ mobilization, ERK activation, and cAMP production, suggesting its potential regulatory role in NE release (Prossnitz et al., 2008).

Studies have shown that the loss of LC-NE neurons and impaired hippocampal glycogen metabolism observed in AD patients are closely associated with cerebral energy metabolic disturbances. Therefore, restoring normal LC-NE function and ameliorating cerebral glycogen metabolic dysfunction are of critical importance for AD treatment. In the present study, we performed a pharmacodynamic evaluation of apigenin in D-galactose-induced cognitive impairment model mice. Following confirmation of its efficacy, we employed untargeted metabolomics to profile metabolic alterations in the mouse hippocampus and conducted pathway enrichment analysis. On this basis, using mouse hippocampal tissues and PC12 cells as research models, we investigated the mechanisms by which apigenin promotes NE release. Furthermore, we utilized targeted metabolomics to explore the relationship between apigenin-induced NE release from noradrenergic neurons and lactate production pathways in astrocytes.

## 2 Materials and methods

### 2.1 Materials

Apigenin (A106676) and chromatographic grade formic acid (F112034) were obtained from Shanghai Aladdin Biochemical Technology Co., Ltd. (Shanghai, China). D-Galactose (D796635) was purchased from Shanghai Macklin Biochemical Technology Co., Ltd. (Shanghai, China). Sodium carboxymethyl cellulose was sourced from Tianjin Guangfu Reagent Co., Ltd. (Tianjin, China). Chromatographic grade methanol (AH230-4) and acetonitrile (AH015-4) were procured from Honeywell International Inc. (Muskegon, MI, United States).

G1 (HY107216), G15 (HY103449), D609 (HY70072), norepinephrine (HY13715), 2-DG (HY13966), and CP-316819 (HY108615) were supplied by MedChemExpress (Monmouth Junction, NJ, USA). L-Lactate (B21929), pyruvate (B24354), UDP-glucose (UDPG, B27933), 3-phosphoglycerate (V30098), glucose-1-phosphate (S11026), glucose-6-phosphate (S11024), and fructose-6-phosphate (S11017) were obtained from Shanghai Yuanye Biotechnology Co., Ltd. (Shanghai, China). Dimethyl sulfoxide (DMSO, D8371) was sourced from Beijing Solarbio Science & Technology Co., Ltd. (Beijing, China). Ammonium acetate (A112057) and ammonia solution (chromatographic grade, A112084) were purchased from Shanghai Aladdin Biochemical Technology Co., Ltd. Cell viability was assessed using the Cell Counting Kit-8 (CCK-8, C0038, Beyotime Biotechnology, Shanghai, China). Intracellular Ca^2+^ levels were detected using the Fluo-4 Calcium Assay Kit (S1061S, Beyotime). Hematoxylin and eosin (H&E) staining was performed using an H&E Staining Kit (G1120, Beijing Solarbio), and Nissl staining was conducted using Nissl Staining Solution (C0117, Beyotime). Glycogen was visualized using a Glycogen PAS Staining Kit (G1360, Beijing Solarbio). Protein concentrations were determined using the BCA Protein Assay Kit (P0010, Beyotime). Phosphate-buffered saline (PBS, 1×, SH30256.01) was purchased from HyClone. SDS-PAGE gel preparation was performed using a SDS-PAGE Denaturing Acrylamide Gel Rapid Preparation Kit (C631100-0200, Sangon Biotech, Shanghai, China). Tris-Glycine SDS Buffer (10×, pH 8.3, CW2566M), Tris-Glycine Transfer Buffer (10×, pH 8.3, CW0044S), and TBST (10×, pH 8.0, CW0043) were sourced from Beijing CoWin Biosciences Co., Ltd. (Beijing, China). The pre-stained protein marker (CW841M) was also obtained from CoWin Biosciences. Immunodetection was performed using the Super Enhanced ECL Chemiluminescence Kit (P0018AS, Beyotime). Cell lysis was carried out using RIPA Lysis Buffer (Strong, P0013B, Beyotime).

### 2.2 Animals

A total of 84 male KM mice (8-week-old, SPF grade) were obtained from SPF Biotechnology Co., Ltd. (Beijing, China), animal license number: SCXK (Beijing) 2024-0001. Animals were housed under controlled conditions (23 °C ± 3 °C, 50% ± 10% humidity, 12 h light/dark cycle) with *ad libitum* access to food and water. After 1 week of adaptation to the environment, the formal experiment was carried out. All feeding and animal experiments were approved by the Animal Ethics Committee of the Yunnan Branch of the Institute of Medicinal Plants, Chinese Academy of Medical Sciences (2024008).

### 2.3 Grouping and treatment

KM mice were randomly divided into Control group (ddH_2_O), D-gal group (125 mg/kg/d), D-gal + Apigenin group (40 mg/kg). The control group was given intraperitoneal injections of physiological saline. The remaining two groups were subjected to intraperitoneal administration of D-galactose (D-gal) at 125 mg/kg per day over an 8-week period to establish the model. Meanwhile, apigenin (Api) treatment commenced from the first week of modeling, with daily oral gavage at 40 mg/kg continuing for the entire 8 weeks and then behavioral experiments were performed.

KM mice were randomly divided into Control (double-distilled water by gavage + saline i.p.), D-gal (double-distilled water by gavage + D-galactose 125 mg/kg i.p.), Api (Api 40 mg/kg by gavage + D-gal 125 mg/kg i.p.), G1 (G1 5 μg/kg s.c. + D-gal 125 mg/kg i.p.), and Api + G15 (Api 40 mg/kg by gavage + G15 185 μg/kg s.c. + D-gal125 mg/kg i.p.). Api was freshly dissolved in 0.5% carboxymethylcellulose sodium (CMC-Na), whereas G1/G15 were freshly dissolved in saline.

### 2.4 Behavioral experiment

Novel Object Recognition (NOR), Morris water maze (MWM) and Y maze were used to evaluate the effects of D-gal on cognition, learning and memory in mice. The behavioral experiments were performed according to previously described protocols (Nasir et al., 2026; Younas et al., 2026).

### 2.5 Sample collection and preparation

24 h after behavioral testing, the mice were anesthetized with isoflurane (5% induced, 2% maintained), and the brain tissues were isolated immediately after death. Brain tissues from three mice per group were fixed in 4% paraformaldehyde for H&E staining, Nissl staining, and other histological experiments. The remaining brain tissues were rapidly frozen in liquid nitrogen and stored at −80 °C until analysis.

### 2.6 Hematoxylin-eosin staining (H&E)

Whole mouse brains were fixed with 4% paraformaldehyde (PFA) at 4 °C for 24 h, then dehydrated through a graded ethanol series (70%, 80%, 90%, and 100%). Tissues were cleared in xylene, embedded in paraffin wax, and sectioned coronally at 5 μm thickness using a microtome (Leica, Germany). Hippocampal sections encompassing CA1, CA2, CA3 and dentate gyrus (DG) regions were stained using a standard H&E staining kit according to the manufacturer’s protocol. Stained sections were examined under an Olympus BX43 microscope, with neuronal nuclei appearing blue and cytoplasm staining pink.

### 2.7 Nissl staining

The Nissl bodies in the hippocampus of mice were stained by Nissl staining kit. Tissue sections from dewaxing to dehydration. Garney’s dyeing solution was immersed for 10 min, rinsed with running water, dehydrated, transparent and sealed. Nissl staining solution was immersed for 10 min, rinsed with running water, dehydrated, transparent and sealed. The CA1, CA2, CA3 and DG regions of hippocampus were observed with OLYMPUS microscope. The images were collected and analyzed, and it was found that the Nissl bodies were blue.

### 2.8 Metabolomics sample processing

Specifically, the frozen mouse hippocampal tissues were weighed into an Eppendorf tube, homogenized with cold MeOH: ACN (v: v, 1:1), and added with 10 μL (10 μg mL^−1^) L-2-chlorophenylalanine as the internal standard solution. Subsequently, the mixture was fully swirled and left on ice to precipitate the protein, centrifuged (10,000 g, 4 °C, 15 min), the supernatant was transferred to a new test tube and dried under N_2_ flow. Before LC-MS analysis, the dry sample was redissolved with 100 μL ACN: H_2_O (V/V, 1:1), ultrasound, centrifugation (4 °C, 13,000 rpm, 15 min), and the supernatant was obtained. The supernatant was put into sample bottle and stored at 4 °C for LC-MS analysis.

### 2.9 Metabolomic analysis

#### 2.9.1 Non-target metabolomic

Sample analysis was performed on ACQUITY UPLC I-Class (Waters, United States) the coupled to the Waters Xevo-G3-Q-Tof (Waters, United States) mass detector. Samples were separated on Waters ACQUITY UPLC HSS T3 column (100 mm × 2.1 mm, 1.8 μm, United States). The mobile phase in the positive ion mode consists of 0.1% formic acid aqueous solution (A) and 0.1% formic acid-acetonitrile (B), and the mobile phase in the negative ion mode consists of 0.5 mM ammonium fluoride-water (A) 100% acetonitrile (B). Gradient elution is as follows: 0–1 min, 99% A; 1–8 min, 99%–1% A; 8–10 min, 1% A; 10.0–10.1 min, 1%–99% A; 10.1–12.0 min, 99% A. The flow rate was 0.5 mL min^−1^, and the column temperature was set at 40 °C.The sample size was 6 μL. Electrospray ionization (ESI) was used. ESI+: Capillary voltage was 3.0 kV, cone hole voltage was 40 V, source temperature was 120 °C, solvent removal temperature was 450 °C, solvent removal gas flow was 1000 L·h^−1^, cone hole blowback gas flow was 50 L·h^−1^. ESI−: the capillary voltage was 2.5 kV, the source temperature was 125 °C, and other parameters are the Da, scanning time is 0.2 s, scanning delay is 0.1 s 0.5 mM sodium formate was used for mass axis correction and 200 ng μL^−1^ leucine enkephalin was used for mass correction to ensure the accuracy of MS analysis.

All the mass spectrum data files obtained by the UPLC-Q-TOF-MS system were preprocessed by the Progenesis QI 2.0 software (Waters, United States), including peak matching, peak extraction and normalization. Import the processed data into Ez info for multivariate data analysis. Metabolites were identified using m/z (error ± 5 ppm), retention time, and mass spectrometry libraries. The metabolic profile of rat brain tissue was constructed using PCA and OPLS-DA. In addition, different variables between the data of each group were found by selecting QC Coefficient of Variation (CV) < 30%, VIP >1, Anova p-value<0.05 and other conditions. The abundance of KEGG pathways was analyzed using MetaboAnalyst 5.0 software (https://www.metabanalyst.ca/) for potential biomarker screening.

#### 2.9.2 Targeted metabolomic

Authentic reference standards for targeted metabolomics analysis included L-lactate (B21929), pyruvate (B24354), UDP-glucose (UDPG, B27933), fructose-6-phosphate (F-6-P, S11017), 3-phosphoglycerate (3-PG, V30098), glucose-1-phosphate (G-1-P, S11026), and glucose-6-phosphate (G-6-P, S11024), all obtained from Shanghai Yuanye Biotechnology Co., Ltd. L-lactate, Pyruvate, UDPG, F-6-P, 3-PG, G-1-P, G-6-P were measured at 2 mg. They were placed in 2 mL volumetric bottles, dissolved with chromatograph-grade methanol and fixed volume, and 1 mg/mL single standard solution was obtained for use. The solution is prepared and stored in the refrigerator at −80 °C. Sample analysis was performed on the Waters ACQUITY UPLC HClass (Waters, USA) coupled to the Triple Quad™ 5500 ± QTRAP™ Read mass spectrometer (AB sciex, United States). The samples were separated on a Waters ACQUITY UPLC HSS T3 column (100 mm × 2.1 mm, 1.8 μm, United States). The mobile phase consists of 0.1% formic acid aqueous solution (A) and 0.1% formic acid-acetonitrile (B), and the gradient elution is as follows: 0–0.5 min, 90% A; 0.5–6 min, 90%–10% A; 6–6.5 min, 10%–90% A; 6.5–10 min, 90% A. The flow rate was 0.3 mL min^−1^, the column temperature was set at 40 °C, and the sample size was 5 μL. Electrospray ionization source (ESI) was used. Multi-reactive ion monitoring (MRM) was used for quantitative analysis. In the positive ion mode, the IonSpray Voltage (IS) was 5.5 kV, and the Temperature (TEM) was 550 °C, Ion Source Gas1 (GS1) pressure was 50 psi, Ion Source Gas2(GS2) pressure was 50 psi; Curtain Gas (CUR) pressure was 35 psi; The Collision Cas (CAD) was 9 and the Dwell time is 50 ms. The mass spectrum parameters were optimized as follows: Phe (166.2 → 120.1, DP: 51 V, CE: 17 V); Tyr (182.4 → 136.3, DP: 58 V, CE: 19 V); DA (154.0 → 137.0, DP: 45 V, CE: 15 V); DOPA (197.9 → 152.1, DP: 60 V, CE: 60 V); NE (170.3 → 152.0, DP: 34 V, CE: 12 V); HVA (183.0 → 105.0, DP: 58 V, CE: 22 V).

### 2.10 Molecular docking

Through database search and literature review, we found that G Protein-Coupled Estrogen Receptor 30 (GPR30) play a key role in the anti-AD of Api. In order to further determine whether the composition of Api in LC had binding ability with these key targets, we conducted molecular simulation with AutoDock and PymoL software to determine its binding ability.

### 2.11 Protein extraction and Western blotting

For brain tissue protein extraction, T-PER Tissue Protein Extraction Reagent (Thermo Scientific) was chosen to isolate the total protein. Radio immunoprecipitation assay (RIPA) buffer and phenylmethanesulfonyl fluoride (PMSF) (Beyotime) were used to extract total cell protein. For secreted protein, after the cells were cultured with serum-free medium for 24 h, the serum-free medium was collected and centrifuged. Added 1 volume of 100% Trichloroacetic acid (TCA) to 4 volumes of the serum-free medium to precipitate secreted protein. Washed protein pellet with cold acetone 3 times before dissolved. Western blot assays were performed to detect the relative protein expression. Briefly, denatured protein was separated via polyacrylamide gelelectrophoresis and then transferred to polyvinylidene fluoride (PVDF) membrane. The membranes were blocked with 5% defatted milk and incubated with primary antibody at 4 °C overnight. Thereafter, the membranes were incubated with horseradish peroxidase-labeled secondary antibodies. The following primary antibodies were used: recombinant anti-GPR30 antibody (ab260033), recombinant anti-PLCβ1 antibody (ab182359), and recombinant anti-Syt1 antibody (ab302627), all from Abcam (Cambridge, United Kingdom); and anti-β-actin antibody (BM3873) from Wuhan Boster Biological Technology Co., Ltd. (Wuhan, China). Horseradish peroxidase (HRP)-conjugated goat anti-rabbit IgG secondary antibody (BA1054) was obtained from Wuhan Boster Biological Technology Co., Ltd.

### 2.12 Cell culture

PC12 cells and primary astrocytes were cultured in DMEM medium (C11995500BT, Gibco, Grand Island, NY, United States) supplemented with 10% fetal bovine serum (FBS, 11011-8611, Zhejiang Tianhang Biotechnology Co., Ltd., Hangzhou, China) and 1% penicillin-streptomycin solution (SV30010, HyClone, Logan, UT, United States). Cells were maintained at 37 °C in a humidified atmosphere containing 5% CO_2_. For subculture, cells were detached using trypsin-EDTA solution (T1320, Beijing Solarbio).

### 2.13 Cell counting kit-8 (CCK-8) assay

Add the CCK-8 reagent to the 96-well culture plate seeded with 1 × 10^5^ MRC-5 cells. which had been treated with different doses of JFG for 24 h, and incubated for 30 min at 37 °C. Absorbance was recorded at 450 nm.

### 2.14 Cellular thermal shift assay (CETSA)

Based on the molecular docking results, GPR30 and apigenin were selected for CETSA analysis. PC12 cells were cultured to the logarithmic growth phase and harvested. Cells were resuspended in PBS and lysed by repeated freeze-thaw cycles, followed by centrifugation to collect the supernatant. The cell lysates were divided into two aliquots: one was treated with apigenin (final concentration, 1 μM) and the other with an equal volume of DMSO as the vehicle control. After incubation at room temperature for 30 min, each aliquot was further divided into equal portions and heated at a gradient of temperatures (36.9 °C–65.0 °C) for 3 min, followed by cooling on ice. The samples were then centrifuged at 12,000 × g for 20 min at 4 °C to remove precipitated proteins. The supernatants were collected and subjected to Western blot analysis using an anti-GPR30 antibody. The band intensities were quantified and normalized to those of the untreated control at the lowest temperature, and the melting curves were plotted to evaluate the thermal stabilization of GPR30 by apigenin.

### 2.15 Measurement of intracellular calcium concentration

Cells were seeded on glass-bottom culture dishes and loaded with 5 µM Fura-2 acetoxymethyl (AM) ester (Thermo Fisher Scientific) in HEPES-buffered physiological saline solution (HPSS) containing 0.02% Pluronic F-127 to facilitate dye dispersion. Loading was performed at 37 °C for 30 min in the dark, followed by a 15-min de-esterification period in dye-free medium to ensure complete intracellular hydrolysis of the AM ester.

Fluorescence imaging was performed using an inverted spinning-disk confocal microscope (Nikon Ti2-E) equipped with a ×40 oil-immersion objective (NA 1.35). For Fura-2, dual-excitation at 340 nm and 380 nm was achieved using a rapid wavelength-switching monochromator, with emission collected at 510 nm. Images were acquired at a frame rate of 1 Hz with an exposure time of 100 ms per frame.

### 2.16 PAS glycogen staining

PC12 cells were seeded onto sterile glass coverslips at an appropriate density. Following two washes with sterile 1× phosphate-buffered saline (PBS), the cells were fixed with PAS fixative for 10 min, washed under running water, and allowed to air-dry. Periodic acid solution was then applied to the coverslips and incubated in the dark for 10 min, after which the coverslips were rinsed under running water for 5 min. Prior to complete drying, Schiff’s reagent was added and the cells were incubated in the dark for 10 min, followed by another 5-min rinse under running water and air-drying. Nuclei were counterstained with hematoxylin for approximately 30 s, rinsed with running water, and air-dried. The coverslips were subsequently mounted with glycerol and examined under a light microscope.

### 2.17 Data analysis

All data results were analyzed and processed by SPSS 21.0 statistical software, and the data were expressed as mean ± standard error (mean ± SEM). The normality of the distribution and the equality of variances were evaluated using the Shapiro-Wilk and Levene tests, respectively. One-way analysis of variance was used for comparison among groups conforming to normal distribution, and LSD was used for post-test. T-test was used to compare the two samples. For data that did not conform to a normal distribution, the Wilcoxon rank sum test was used for nonparametric analysis. For the Morris water maze escape latency, a repeated-measures two-way ANOVA (group × time) was employed, with Mauchly’s test for sphericity and Greenhouse–Geisser correction when sphericity was violated, followed by Bonferroni *post hoc* test. P < 0.05 indicated statistical significance.

## 3 Results

### 3.1 Api improved cognitive impairment in D-gal model mice *in vivo*

To generate the D-gal-induced cognitive impairment model, we followed the experimental procedure schematized in Figure 1A. Compared with the Control group, mice in the D-gal-treated group exhibited a significant decrease in body weight, which was markedly reversed by apigenin (Api) administration (Figure 1B). Compared with Control group mice, the number of new object recognition index crossing the platform, spontaneous conversion rate (P < 0.05) and target quadrant residence time of mice in D-gal group were reduced. The localization latency was significantly increased (P < 0.01). It was indicated that the model of cognitive impairment induced by LPS has been successfully established. The administration of EZP significantly improved cognitive impairment in mice (Figures 1C–F). In order to clarify the protective effect of Api on hippocampal neurons, we used H&E and Nissl to detect the state of hippocampal neurons in each group of mice. H&E results showed that compared with the Control group, the neurons in the hippocampus of mice in D-gal group had loose arrangement, irregular shape, and obvious nuclear atrophy, among which the neurons in CA1 and CA3 were more seriously damaged (Figure 1G). The results of Nissl showed that compared with the Control group, CA1, CA2 and neuronal cells in the hippocampus of mice in D-gal group were arranged in a looser way, necrotic neurons were increased, the number of nissl bodies was decreased, and nuclear pyknosis was obvious (P < 0.05), while there was no significant difference in neurons in DG region (Figures 1H,I). The neuronal state of hippocampus can be restored after the administration of Api.

**FIGURE 1:**
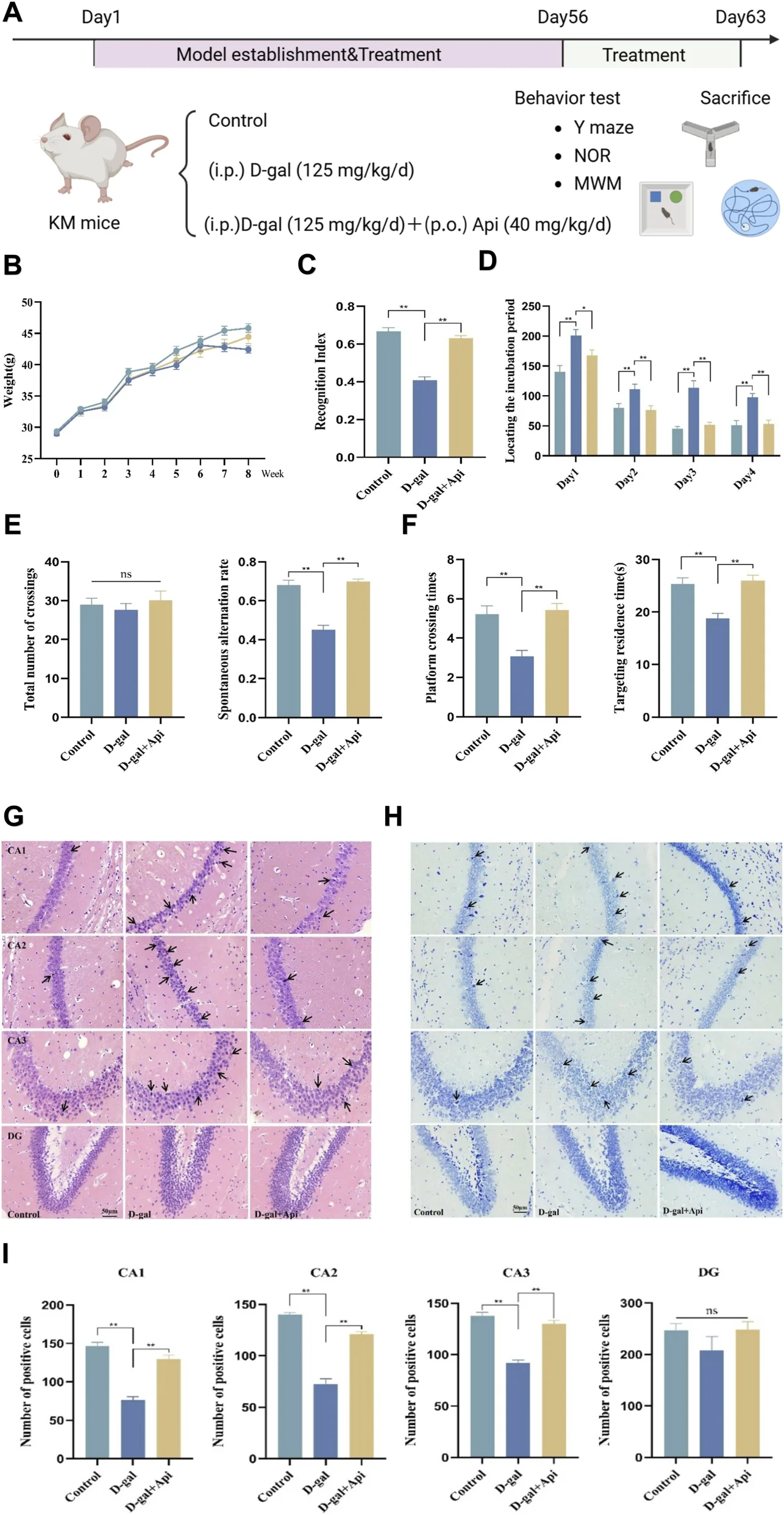
Pharmacodynamic effects of Api on cognitive impairment in D-gal-induced mice. **(A)** Schematic diagram of the experimental procedure. **(B)** Body weight measurement (n = 10). **(C)** Novel object recognition test (n = 10). **(D, E)** Morris water maze test: **(D)** escape latency during the place navigation trial (n = 10), analyzed by repeated-measures two-way ANOVA; **(E)** representative swimming trajectories during the spatial probe test (n = 10). **(F)** Y-maze test (n = 10). **(G)** Pathological morphology of hippocampal neurons (n = 3). **(H)** Nissl body expression in the hippocampus (n = 3). Note: black arrows indicate nuclear shrinkage and necrotic neurons. **(I)** Nissl body expression in the hippocampal CA1, CA2, CA3, and dentate gyrus (DG) regions (n = 3). Data are presented as mean ± SEM. ns P > 0.05, *P < 0.05, **P < 0.01.

### 3.2 Api improved the metabolic profile of D-gal model and regulates NE biosynthesis

Due to the critical role of LC degeneration in the occurrence and progression of AD, we focused on the LC tissue of mice in each group to investigate the mechanism by which Api improves cognitive impairment in D-gal model mice from a metabolic perspective. First, nontargeted metabolomics studies were conducted using UPLC-Q-TOF-MS/MS to obtain data reflecting the sample information of each group and the metabolic profile chromatogram of LC tissue (Figure 2A). PCA and OPLS-DA were employed for pattern recognition and visual analysis of the mass spectrum data from the Control and D-gal groups. As shown in Figures 2B,C the Control group and D-gal group were significantly separated in both positive and negative ion scanning modes. The Permutation test confirmed that Q2 and Y2 met the required criteria, indicating the model had strong explanatory power without overfitting. These results demonstrated that D-gal induction significantly altered endogenous metabolites in LC tissue, confirming successful model establishment. Using the above analytical methods, we identified 15 differentially expressed metabolites in mouse LC tissue by comparing with control products, literature, and database searches (Metlin, HMDB) (Table 1; Figure 2D).

**FIGURE 2:**
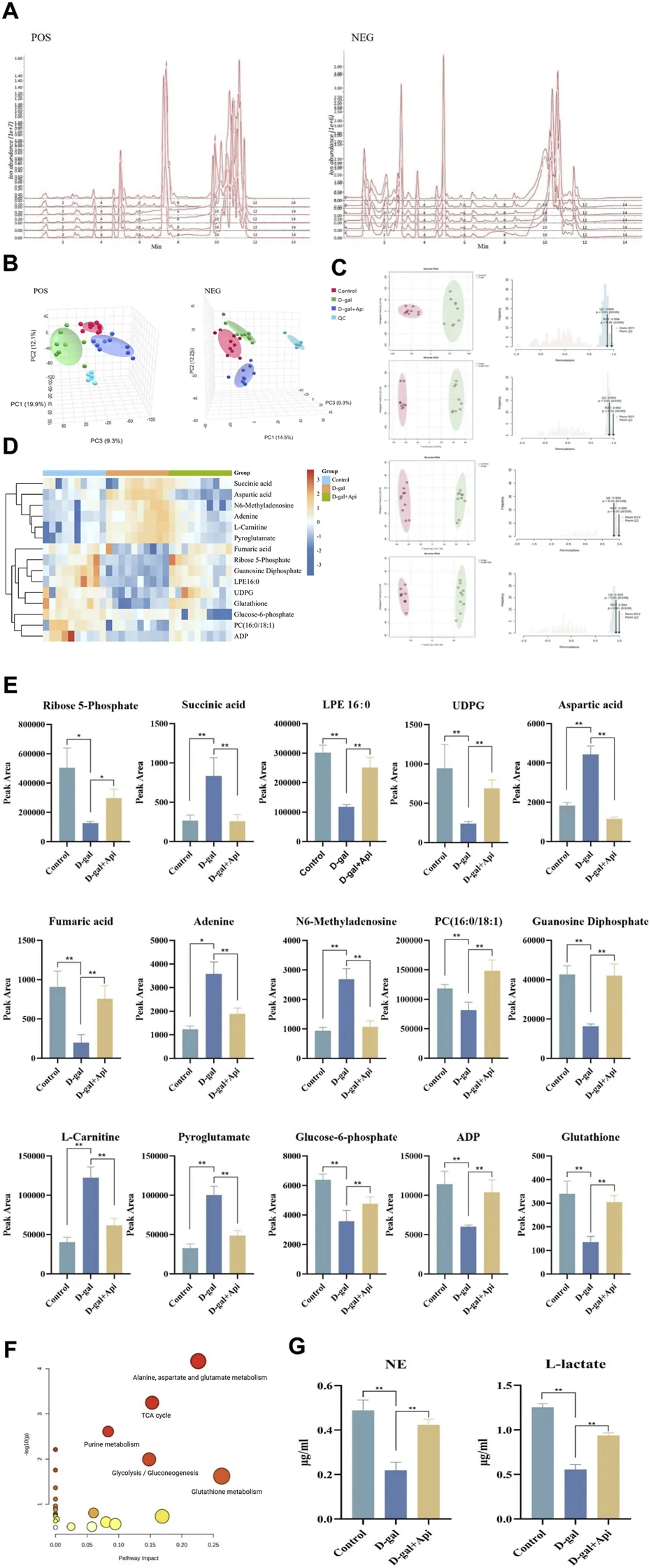
Metabolomic analysis of hippocampal tissue in D-gal-induced mice treated with Api. **(A)** QC sample Metabolic profile. **(B)** 3D-PCA. **(C)** OPLS-DA. **(D)** Heatmap of differential metabolites. **(E)** Enrichment analysis of differential metabolites (n = 6). **(F)** Key metabolites of NE anabolic pathway. **(G)** NE and L-lactate levels in mouse hippocampus (n = 6). *P < 0.05, **P < 0.01.

**TABLE 1:** Differential metabolite information in mouse LC tissue.

No.	RT (min)	Adducts	m/z	Formular	Identification	D-gal vs.Control	Api vs. D-gal
1	1.037	[M − H]−	229.0115	C_5_H_11_O_8_P	Ribose 5-phosphate	↓*	↑^#^
2	3.43	[M + H]+	136.0632	C_5_H_5_N_5_	Adenine	↑*	↓^##^
3	2.988	[M + H]+	282.1197	C_11_H_15_N_5_O_4_	N6-methyladenosine	↑**	↓^##^
4	2.305	[M − H]−	306.3066	C_10_H_17_N_3_O_6_S	Glutathione	↓**	↑^##^
5	10.374	[M + Na]+	782.5689	C_42_H_82_NO_8_P	PC(16:0/18:1)	↓**	↑^##^
6	1.963	[M + H]+	444.0334	C_10_H_15_N_5_O_11_P_2_	Guanosine diphosphate	↓**	↑^##^
7	1.026	[M + H]+	162.1117	C_7_H_15_NO_3_	L-carnitine	↑**	↓^##^
8	0.978	[M + H]+	130.049	C_5_H_7_NO_3_	Pyroglutamate	↑**	↓^##^
9	2.693	[M − H]−	117.0188	C_4_H_6_O_4_	Succinic acid	↑**	↓^##^
10	11.355	[M + H]+	261.2248	C_6_H_13_O_9_P	Glucose-6-phosphate	↓**	↑^##^
11	2.843	[M − H]−	452.2689	C_21_H_44_NO_7_P	LPE16:0	↓**	↑^##^
12	4.684	[M − H]−	566.0552	C_15_H_24_N_2_O_17_P_2_	UDPG	↓**	↑^##^
13	1.118	[M − H]−	115.0023	C_4_H_4_O_4_	Fumaric acid	↓**	↑^##^
14	1.937	[M + H]+	428.0375	C_10_H_15_N_5_O_10_P_2_	Adenosine diphosphate	↓**	↑^##^
15	2.018	[M − H]−	132.0283	C_4_H_7_NO_4_	Aspartic acid	↑**	↓^##^

*P < 0.05, **P < 0.01 vs. the Control group; #P < 0.05, ##P < 0.01 vs. the D‐gal group.

Compared with the Control group, the levels of nine metabolites, including glucose-6-phosphate, citric acid, ribose 5-phosphate, and ADP, were markedly decreased in the hippocampal tissues of D-gal-treated mice, whereas six metabolites, including aspartic acid, pyroglutamate, and glutathione, were significantly increased. Notably, these differential metabolites were substantially reversed following Api administration in Figure 2E.

To further elucidate the alterations in metabolic pathways within the hippocampal tissues of D-gal-induced model mice, pathway enrichment analysis was performed on the potential biomarkers that were rescued by Api treatment. As shown in Figure 2E, the enriched pathways primarily involved alanine, aspartate and glutamate metabolism, purine metabolism, and glutathione metabolism, as well as multiple energy metabolism pathways, including the TCA cycle, glycolysis/gluconeogenesis, and the pentose phosphate pathway. These findings suggest that the neuroprotective effects of apigenin against D-gal-induced injury may be associated with the regulation of energy metabolism pathways in Figure 2F.

Api treatment significantly upregulated the levels of UDP-glucose (UDPG) and glucose-6-phosphate (G6P), two key metabolites involved in glycogen metabolism and the glycolytic pathway, the end product of which is lactate. To further investigate whether the neuroprotective effects of Api are associated with NE and L-lactate levels, targeted metabolomics analysis was performed to assess NE and L-lactate levels in the hippocampus. As shown in Figure 2G, the D-gal group exhibited decreased L-lactate levels and decreased NE levels compared to the Control group (P < 0.01). In contrast, the D-gal + Api group showed elevated NE levels and L-lactate levels (P < 0.01). Api may exert neuroprotective effects by modulating NE release, which in turn promotes lactate production in hippocampal astrocytes, ultimately influencing the astrocyte-neuron lactate shuttle (ANLS) and ameliorating energy metabolism deficits in hippocampal neurons in D-gal-induced cognitive impairment.

### 3.3 Apigenin attenuates cognitive deficits in D-galactose-induced aging mice via GPR30

Our previous studies have established, on the basis of pharmacodynamic assays and molecular docking, that GPR30 is a potential target for the pharmacological actions of apigenin. Furthermore, we have shown that apigenin increases NE levels and L-lactate levels in the hippocampus of D-gal-induced mice. Nevertheless, how apigenin regulates NE release via GPR30 remains to be fully elucidated.

According to the experimental workflow shown in Figure 3A, group allocation was performed accordingly. Compared with the D-gal-treated group, all treatment groups exhibited a relatively smaller decrease in body weight in Figure 3B. The D-gal + Api and D-gal + G1 groups showed significant increases in the novel object recognition index, the number of platform crossings, spontaneous alternation rate (P < 0.01), and the time spent in the target quadrant, along with a marked reduction in escape latency (P < 0.01), indicating that both GPR30 activation and apigenin exert neuroprotective effects. Furthermore, compared with the D-gal + Api group, the D-gal + Api + G15 group exhibited significant decreases in the novel object recognition index, platform crossings, spontaneous alternation rate (P < 0.01), and target quadrant time, accompanied by a significant prolongation of escape latency (P < 0.01), suggesting that GPR30 inhibition attenuated the neuroprotective effects of apigenin (Figures 3C–F).

**FIGURE 3:**
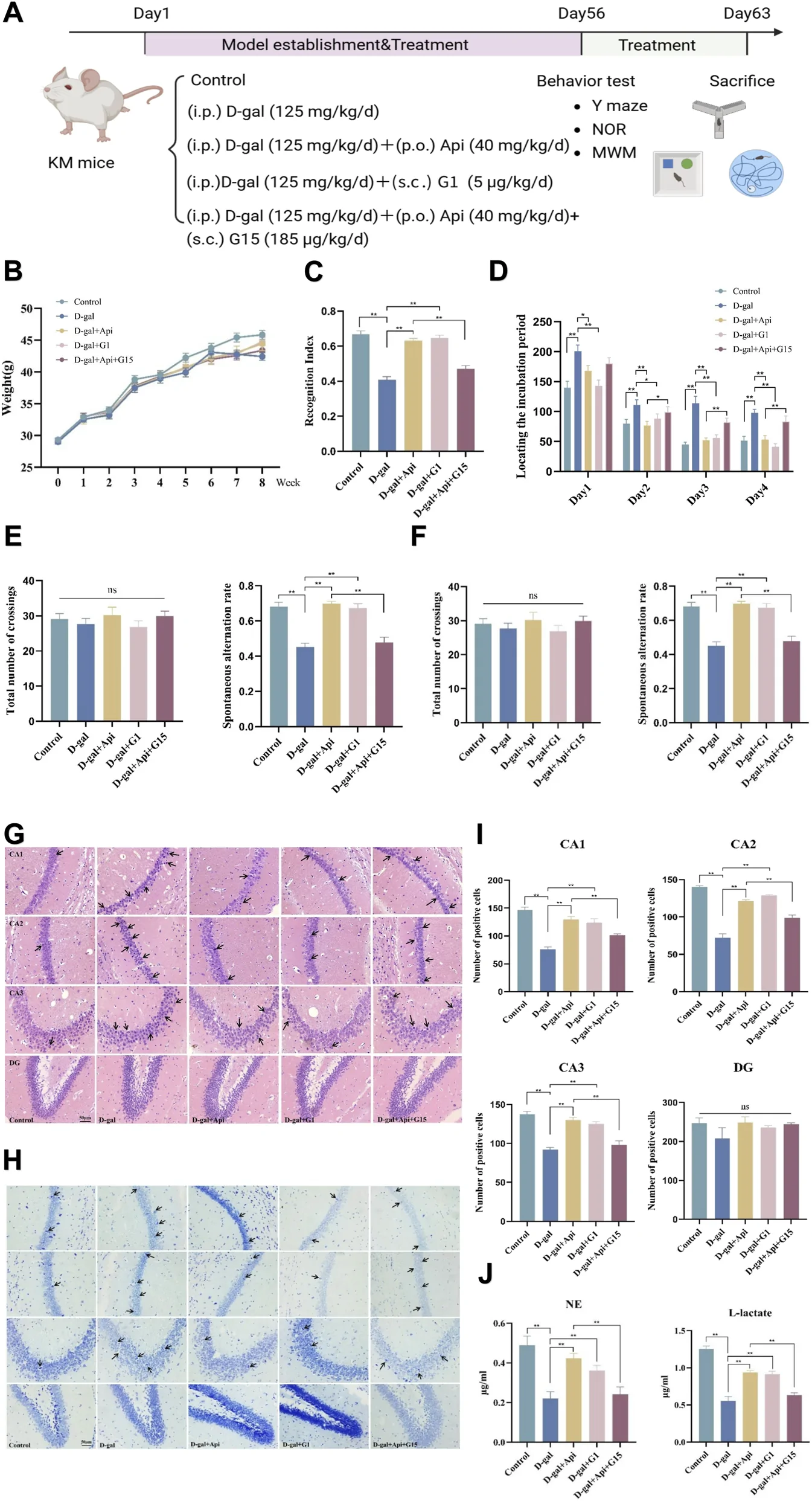
Pharmacodynamic study of apigenin combined with g15 on cognitive dysfunction in the D-galactose-induced mouse model. **(A)** Schematic diagram of experimental procedure. **(B)** Body weight measurement (n = 10). **(C)** New object recognition experiment (n = 10). **(D, E)** Water maze experiment, **(D)** escape latency during the place navigation trial (n = 10), analyzed by repeated-measures two-way ANOVA; **(E)** Representative trajectory of mouse swimming, spatial probe test (n = 10). **(F)** Y maze experiment (n = 10). **(G)** Pathological morphology of nerve cells in hippocampus (n = 3). **(H)** Expression of nissl bodies in hippocampus. Note: black arrow indicates nuclear shrinkage and necrotic neuron cell (n = 3). **(I)** Nissl body expression in the hippocampal CA1, CA2, CA3, and dentate gyrus (DG) regions (n = 3). **(J)** NE and L-lactate levels in mouse hippocampus (n = 6). ns P > 0.05, *P < 0.05, **P < 0.01.

H&E staining was performed to evaluate neuronal morphology. Compared with the Control group, the D-gal-treated group exhibited loosely arranged hippocampal neurons with irregular morphology and pronounced nuclear atrophy. In contrast, the D-gal + Api and D-gal + G1 groups showed marked morphological improvements across all hippocampal subregions. However, compared with the D-gal + Api group, the D-gal + Api + G15 group displayed deteriorated neuronal morphology in Figure 3G.

Nissl staining further revealed that, compared with the Control group, the D-gal-treated group exhibited loosely arranged neurons with increased necrotic neurons, a significant reduction in Nissl body counts (P < 0.01), and pronounced karyopyknosis in all hippocampal subregions except the dentate gyrus (DG), where no significant neuronal damage was observed. Compared with the D-gal group, the D-gal + Api and D-gal + G1 groups showed markedly improved neuronal morphology across hippocampal subregions, characterized by regularly arranged neurons with clear and intact boundaries, along with significantly increased Nissl body counts (P < 0.01). However, compared with the D-gal + Api group, the D-gal + Api + G15 group exhibited poorer neuronal morphology and reduced Nissl body counts across all hippocampal subregions (P < 0.01) in Figures 3H,I.

As shown in Figure 3J, compared with the Control group, the levels of norepinephrine (NE) and lactate in the hippocampal tissues of the D-gal-treated group were significantly decreased (P < 0.01). Administration of apigenin (Api) and G1 significantly elevated the levels of NE and lactate (P < 0.01). However, compared with the D-gal + Api group, the D-gal + Api + G15 group exhibited significantly suppressed levels of hippocampal NE and lactate (P < 0.01). The neuroprotective effects of apigenin were largely abrogated by GPR30 antagonism with G15. Collectively, the neuroprotective effects of apigenin against D-galactose-induced injury are primarily dependent on the GPR30 target.

### 3.4 Apigenin promotes NE release in PC12 cells through the GPR30-mediated PLCβ1-Ca^2+^-Syt1 signaling pathway

However, given the cellular heterogeneity of hippocampal tissue *in vivo*, it is difficult to precisely dissect the underlying molecular mechanisms using animal models alone. Therefore, we performed *in vitro* experiments using PC12 cells (a rat adrenal pheochromocytoma cell line) for further mechanistic exploration. PC12 cells are a well-established neuroendocrine cell model that expresses norepinephrine transporters and synapse-associated proteins, and their vesicle fusion machinery is largely comparable to that of primary neurons. Accordingly, this cell line has been widely employed as a surrogate model for studying neurosecretion (Chen and Wang, 2023). In the present study, we therefore adopted PC12 cells to investigate the molecular mechanisms underlying the regulation of NE release by apigenin (Kumar et al., 1998).

To determine the optimal concentration of D-gal for establishing an *in vitro* injury model, PC12 cells were treated with varying concentrations of D-gal (0–60 mg/mL) for 24 h. As shown in Figure 4A, the optimal concentration was determined to be 60 mg/mL, at which the cell viability was maintained at approximately 45.2%. The optimal concentration of apigenin was determined to be 1 μM, at which the cell viability was increased to 76.5%, significantly reversing the D-gal-induced reduction in cell viability in Figure 4B (P < 0.01). Therefore, 1 μM Api was selected as the subsequent dose. To evaluate the binding affinity between GPR30 and apigenin, cellular thermal shift assay (CETSA) was performed. As shown in Figures 4C,D, the GPR30 protein in the control group underwent denaturation and inactivation at 59.7 °C. In contrast, the apigenin-treated group exhibited enhanced thermal stability of GPR30, as evidenced by an elevated melting temperature (Tm), suggesting that apigenin may form a stable complex with GPR30 and supporting potential target engagement. The results demonstrated that Api binds to the protein target through visible hydrogen bonds and strong electrostatic interactions, with a binding energy of −7.7 kcal/mol, suggesting favorable binding to GPR30 in Figure 4E. Next, we examined the protein expression levels of Ca^2+^ signaling-related proteins, with a focus on GPR30, PLCβ1, and Syt1. Compared with the D-gal group, the protein expression levels of GPR30, PLCβ1, and Syt1 were significantly increased in the D-gal + Api group (P < 0.01). However, compared with the D-gal + Api group, administration of the GPR30 antagonist G15 significantly decreased the expression of GPR30, PLCβ1, and Syt1 (P < 0.01). Furthermore, treatment with D609 (a specific PLC inhibitor) significantly reduced the protein levels of PLCβ1 and Syt1 (P < 0.05), while GPR30 expression exhibited a decreasing trend without statistical significance in Figures 4F–I. Meanwhile, as shown in Figure 4J, Ca^2+^ fluorescence staining revealed that, compared with the Control group, the intracellular Ca^2+^ fluorescence intensity was markedly decreased in the D-gal group. However, apigenin treatment significantly enhanced the fluorescence intensity. Notably, the fluorescence intensity in the D-gal + Api + G15 and D-gal + Api + D609 groups were attenuated compared with the D-gal + Api group. Consistent with the previous experiments, NE levels were measured, and it was found that compared with the D-gal + Api group, NE levels in the D-gal + Api + G15 and D-gal + Api + D609 groups were significantly decreased (P < 0.01) in Figure 4K. The above findings suggest that apigenin promotes NE release through the GPR30-mediated PLCβ1-Ca^2+^-Syt1 signaling pathway. We further speculate that the released NE may subsequently act on β_2_-AR on the astrocytic membrane on the astrocytic membrane to promote lactate production.

**FIGURE 4:**
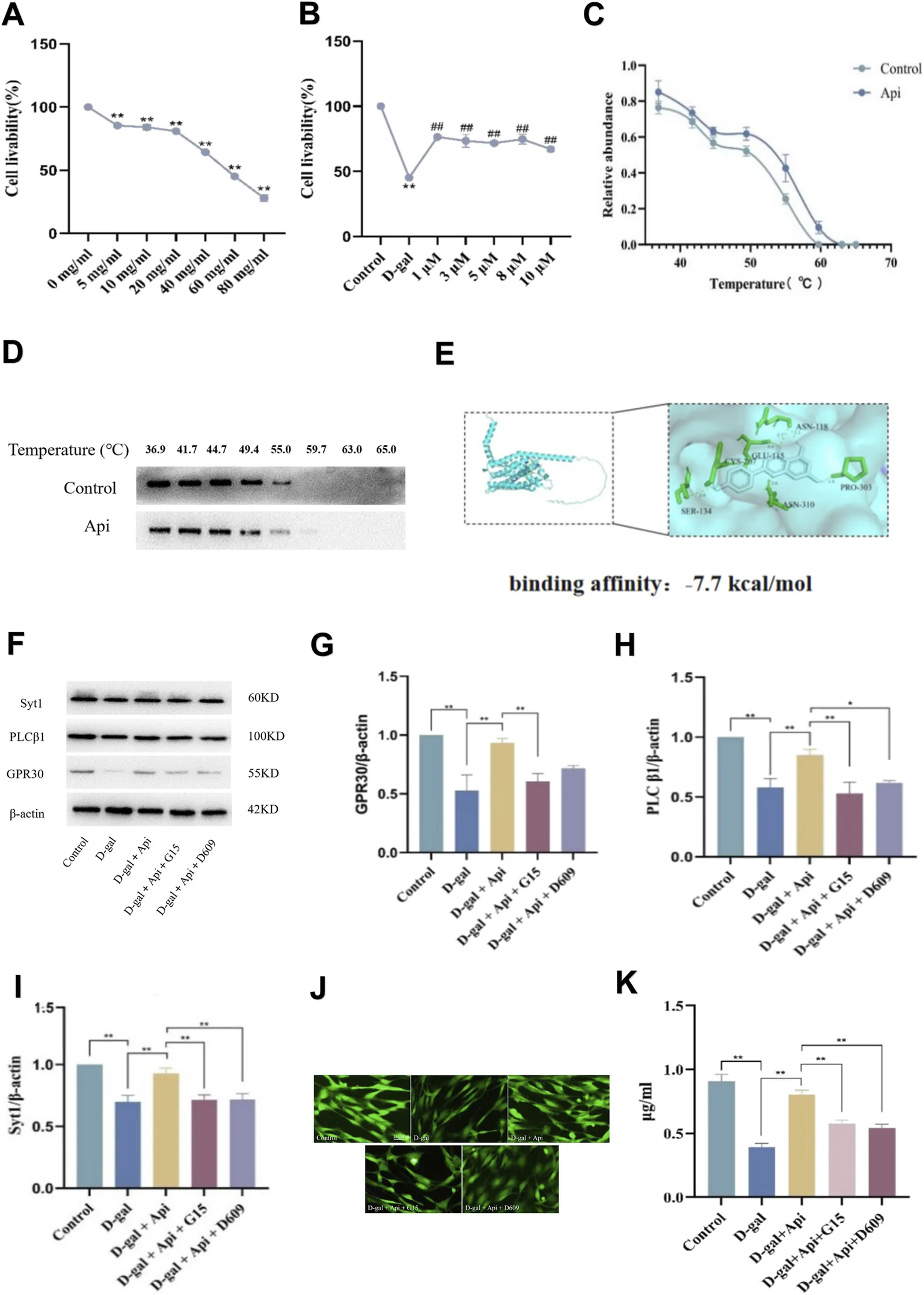
**(A)** Screening of D-gal effective concentration in PC12 cell line. **(B)** Screening of Api effective concentration in PC12 cell line. Data are presented as mean ± SD. **P < 0.01 versus Control group; ##P < 0.01 versus D-gal group. **(C)** The interaction between the target protein GPR30 and Api in PC12 cells was confirmed by CETSA test, and the change of the remaining soluble protein with temperature was detected. The experimental results were represented by specific protein bands. DMSO treatment was a negative control. **(D)** CETSA melting curve. Temperature is a function curve in horizontal coordinates and the relative strength of specific bands is a function curve in vertical coordinates, which represents the trend of the soluble protein amount of the target protein changing with temperature, which is called the apparent melting curve. **(E)** Molecular docking simulation. **(F)** Immunoblotting of GPR30, PLC β1, Syt1 in each group. **(G–I)** Quantification of GPR30, PLC β1, Syt1 in each group. **(J)** Effect of apigenin on Ca^2+^ level in D-gal induced PC12 cells. **(K)** NE level in PC12 cell culture medium (n = 3). ns P > 0.05, *P < 0.05, **P < 0.01.

### 3.5 Apigenin promotes glycogenolysis in astrocytes and elevates lactate levels by facilitating NE secretion from neurons

To determine the optimal concentrations of D-galactose and apigenin in astrocytes, we performed dose-response viability assays. As shown in Figures 5A,B, astrocytes were treated with graded concentrations of D-gal (0–100 mg/mL) or apigenin (0–8 μM), and cell viability was assessed by CCK-8 assay. The optimal concentrations were selected for subsequent mechanistic studies. As shown in Figure 5A, the optimal concentration of D-galactose for inducing injury in astrocytes was determined to be 60 mg/mL, at which cell viability was maintained at approximately 59.6%. Furthermore, as presented in Figure 5B, the optimal concentration of apigenin was found to be 0.1 μM, which significantly increased cell viability to 86.8% compared with the D-gal group (P < 0.01). Subsequent PAS staining (Figure 5C) revealed that astrocytes in the Control group exhibited strong purplish-red staining, indicating abundant and uniformly distributed glycogen. In contrast, the D-gal group showed markedly reduced glycogen storage, with negligible purplish-red coloration. However, treatment with Api or NE significantly improved glycogen levels in both groups, as evidenced by enhanced staining intensity. Notably, co-treatment with the hexokinase inhibitor 2-DG attenuated the NE-induced increase in glycogen levels, whereas administration of the glycogen phosphorylase inhibitor CP-316819 further intensified the glycogen staining.

**FIGURE 5:**
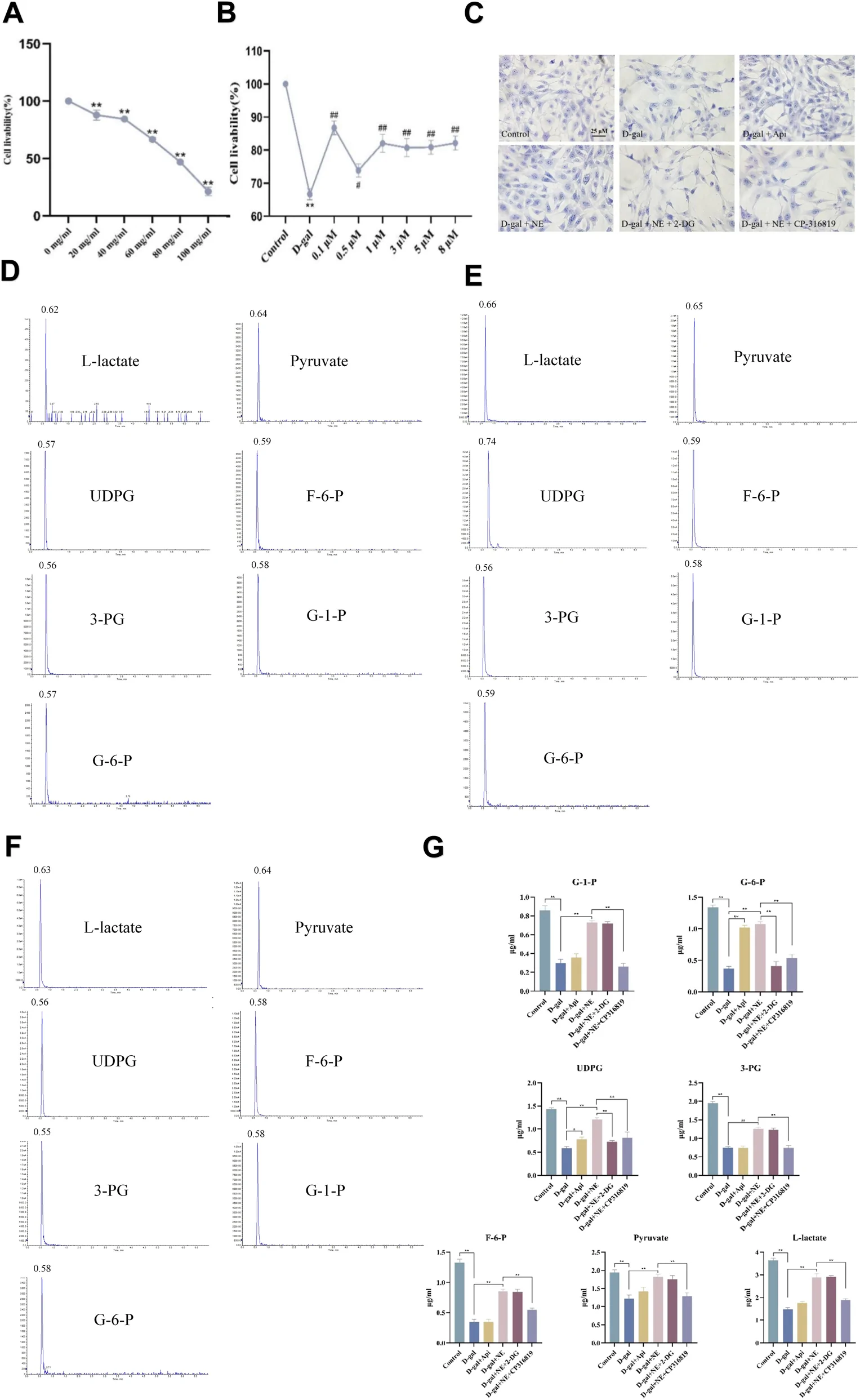
Effects of D-gal and Api on glycogen metabolism and lactate production in astrocytes. **(A, B)** Screening of the optimal concentrations of D-gal and Api in astrocytes (n = 5). **P < 0.01 vs. the Control group; ^#^P < 0.05 and ^##^P < 0.01 vs. the D-gal group. **(C)** Glycogen PAS staining results (400×) (n = 3). **(D)** Representative chromatogram of the blank cell sample. **(E)** Chromatogram of the simulated cell sample. **(F)** Chromatogram of the reference solution. **(G)** Key metabolites of glycogenolysis and glycolysis pathways (mean ± SEM, n = 8). *P < 0.05, **P < 0.01.

To further investigate how apigenin-induced elevation of neuronal NE affects astrocytic lactate levels, we performed targeted metabolomics analysis to comprehensively explore the sources of lactate production in astrocytes. The PAS staining results demonstrated that both apigenin and NE increased glycogen synthesis in astrocytes. This effect may be attributed to the activation of distinct signaling pathways: apigenin may act through GPR30 receptors on astrocytes to activate the PI3K-Akt-GSK3β signaling cascade, whereas NE may activate the cAMP/PKA/CREB pathway, both of which converge to enhance glycogen synthase (GS) activity and promote glycogen synthesis. As shown in Figures 5D–F, the standard chromatographic peaks of each analyte were consistent with those in the blank cell sample solution, with no additional peaks detected, indicating that the detection of each analyte was free from interference. As shown in Figure 5G, compared with the Control group, the levels of L-lactate, pyruvate, UDPG, F-6-P, 3-PG, G-1-P, and G-6-P were decreased in the D-gal group. Relative to the D-gal group, apigenin treatment did not significantly restore L-lactate levels, whereas NE treatment significantly increased L-lactate and pyruvate levels (P < 0.01). Furthermore, compared with the D-gal + NE group, co-treatment with 2-DG (a hexokinase inhibitor) significantly reduced G-6-P and UDPG levels (P < 0.01), with no obvious changes in the other metabolites. In contrast, treatment with CP-316819 (a glycogen phosphorylase inhibitor) significantly decreased all of the above metabolites compared with the D-gal + NE group (P < 0.01). These results indicate that apigenin alone did not significantly promote lactate production in astrocytes, whereas NE treatment significantly increased lactate levels.

## 4 Discussion

D-gal is commonly used to establish animal and cellular models of aging and aging-related cognitive impairment, as it induces and accelerates aging (Zhou et al., 2015). This model induces learning and memory impairments in mice, primarily manifested as reduced spatial learning and recognition abilities in behavioral tests such as the Morris water maze, Y-maze, and novel object recognition, which resemble the cognitive deficits observed in AD patients (Zhang et al., 2022). In addition, D-gal administration leads to impaired hippocampal neuronal activity and apoptosis in these model mice, which are often assessed by H&E and Nissl staining to evaluate morphological changes and the extent of neuronal damage (Li et al., 2025). Therefore, in the present study, we used the D-gal-induced cognitive impairment mouse model and employed behavioral tests and histomorphological analyses to evaluate the therapeutic efficacy of apigenin.

The locus coeruleus (LC) supplies NE to the hippocampus and regulates various behavioral and cognitive functions. The LC is among the first sites where tau pathology emerges in AD, and studies using APP/PS1 mouse models have shown that LC lesion-induced NE dysregulation exacerbates hippocampal energy metabolic disturbances, neuroinflammation, and cognitive deficits (Jacobs et al., 2021). Our experimental results revealed that hippocampal NE levels were decreased in the D-gal model, whereas apigenin treatment reversed this trend, suggesting that apigenin may promote LC-NE release in the hippocampal region. Furthermore, neuronal energy metabolism in the hippocampus is closely linked to lactate levels. Lactate produced by astrocytes not only meets their own energy demands but also supports neuronal energy metabolism through the ANLS, playing a critical role during periods of high synaptic activity or under pathological conditions. Our results showed that hippocampal lactate levels were reduced in the model group, while apigenin administration increased lactate production, indicating that apigenin may directly or indirectly regulate astrocytic lactate production and thereby ameliorate neuronal energy metabolic deficits (Beard et al., 2021).

NOR test was used to assess recognition memory; the Y-maze and MWM tests were used to evaluate working memory, spontaneous alternation behavior, and spatial learning and memory, respectively. Our results showed that model mice exhibited reduced novel object recognition index, spontaneous alternation rate, and time spent in the target quadrant, along with significantly prolonged escape latency, indicating impaired learning and memory as well as spatial memory deficits. Following apigenin treatment, all behavioral parameters showed improvement. This suggests that apigenin enhanced learning and memory in D-gal-induced cognitive impairment mice and exerted beneficial effects on cognitive function (Hinte et al., 2021).

HPC, a critical structure located in the medial temporal lobe and a key component of the limbic system, is responsible for processing episodic and spatial memory and serves as the site where short-term memory is converted into long-term memory (Liao et al., 2024). In AD patients, the hippocampus is particularly vulnerable to pathological changes and reduced neurogenesis, leading to impairments in object memory, contextual fear conditioning, and spatial learning (Kim et al., 2022). H&E and Nissl staining revealed that in model mice, hippocampal CA1, CA2, and CA3 neurons exhibited loosely arranged morphology, increased karyopyknosis, and decreased Nissl body counts. Apigenin treatment significantly ameliorated these morphological alterations, suggesting its neuroprotective effects.

In the present study, we employed G1 (GPR30 agonist) as a positive control and G15 (GPR30 antagonist) as a blocking agent. Through behavioral tests and histomorphological analyses, we demonstrated that both apigenin and G1 effectively alleviated cognitive deficits in AD model mice, whereas co-administration of G15 significantly attenuated the ameliorative effects of apigenin. Furthermore, hippocampal NE and lactate levels were decreased in the D-gal model group, and these trends were reversed by apigenin and G1 treatment. However, compared with the D-gal + Api group, the D-gal + Api + G15 group exhibited reduced NE and lactate levels, suggesting that apigenin may promote NE release and lactate production through GPR30, thereby improving learning and memory deficits in AD model mice. Nevertheless, given the cellular heterogeneity of hippocampal tissue *in vivo*, we performed *in vitro* experiments using PC12 cells (a rat adrenal pheochromocytoma cell line) for further mechanistic studies. PC12 cells are a well-established neuroendocrine cell model that expresses norepinephrine transporters and synapse-associated proteins, and their vesicle fusion machinery is largely comparable to that of primary neurons; thus, this cell line has been widely employed as a surrogate model for studying neurosecretion (Kumar et al., 1998).

NE synthesis is regulated by TH and DβH, whereas synaptic vesicles play a dual role in NE release-they determine the quantity of NE released at nerve terminals and orchestrate the entire release process. Calcium ions are critically involved in regulating vesicular exocytosis. Key proteins participating in neuronal exocytosis include SNARE proteins, SNARE regulatory proteins, and calcium-binding proteins. Among these, SNARE proteins constitute the core molecular machinery mediating vesicle fusion, while synaptotagmin 1 (Syt1) serves as the Ca^2+^ sensor that triggers vesicle fusion, governing both the priming and fusion of synaptic vesicles (Chanaday et al., 2021). Syt1 is a typical type I membrane protein, and studies have shown that the rapid secretory component of neuronal exocytosis is largely abolished in Syt1-knockout mice. Cytosolic Ca^2+^ in axons can originate from either extracellular Ca^2+^ influx or intracellular Ca^2+^ release from stores such as the endoplasmic reticulum and mitochondria. Inositol 1,4,5-trisphosphate receptors (IP_3_Rs) are critical channel proteins mediating Ca^2+^ release from the endoplasmic reticulum (Gambardella et al., 2021). GPR30, a GPCR, is localized on various membranous structures including synaptic vesicles, endocytic vesicles, and secretory vesicles. Many neurotransmitters and hormones regulate intracellular second messengers (Ca^2+^, IP_3_, and DAG) via GPCR-G protein-mediated transmembrane signaling, indirectly modulating multiple steps of exocytosis to ensure precise spatiotemporal and intensity control (Prossnitz and Barton, 2023).

Phospholipase C (PLC) primarily acts on phosphatidylinositol 4,5-bisphosphate (PIP_2_), hydrolyzing it into inositol 1,4,5-trisphosphate (IP_3_) and diacylglycerol (DAG), both of which play critical roles in intracellular signaling (Nakamura and Fukami, 2017). PLC is classified into several isoforms, including PLCβ, PLCγ, and PLCδ. Among these, PLCβ is a family of enzymes regulated by G protein-coupled receptors and comprises four subtypes: PLCβ1, PLCβ2, PLCβ3, and PLCβ4. PLCβ1 is predominantly expressed in brain tissue, where it functions in neuronal dendrites and axons, and participates in neurotransmitter release and synaptic plasticity at synapses (Kim et al., 1997). In this chapter, we employed G15 and D609 (a PLCβ1/IP_3_R inhibitor) to investigate whether apigenin mediates NE release in PC12 cells through the GPR30-PLCβ1-Ca^2+^-Syt1 signaling axis.

CETSA results showed that apigenin exhibited measurable binding to GPR30, supporting potential target engagement. Western blot analysis further revealed that GPR30 protein expression was increased in the D-gal + Api group, which may be attributed to feedback regulatory mechanisms and activation of other signaling pathways. Apigenin treatment upregulated the protein levels of PLCβ1 and Syt1, as well as intracellular Ca^2+^ levels, whereas these effects were attenuated in the D-gal + Api + G15 and D-gal + Api + D609 groups. Consistent with these observations, NE levels in the culture medium exhibited a similar trend. Collectively, these findings indicate that apigenin promotes NE release through the GPR30-mediated PLCβ1-Ca^2+^-Syt1 signaling pathway. We further speculate that the released NE may subsequently enter astrocytes via β_2_-adrenergic receptors (β_2_-AR) on the astrocytic membrane to promote lactate production.

Astrocytes are predominantly located in the central nervous system (CNS), including the brain, cerebellum, and spinal cord, where they are interposed between neurons and form a complex network in close proximity to neuronal somata and processes (Guillamón-Vivancos et al., 2015). The bioenergetics of brain activity largely depend on the functional and metabolic interactions between neurons and astrocytes (Massucci et al., 2013). Neurons serve as the core units for information processing and transmission in the brain, and their activity demands substantial energy consumption, encompassing processes such as action potential generation, neurotransmitter release, and maintenance of intracellular ion gradients (Azarfar et al., 2018). In AD, neuronal glucose supply is compromised due to pathological factors including impaired transporter function and mitochondrial dysfunction. Moreover, neurons are unable to store glucose in the form of glycogen. Consequently, neurons rely on lactate derived from astrocytes to generate ATP. In astrocytes, lactate is produced from either glycogen or glucose in response to neuronal activity signals (Alberini et al., 2018). Glucose is first phosphorylated to glucose-6-phosphate G-6-P by HK. G-6-P can either be stored as glycogen via GS or enter glycolysis to produce pyruvate, which is subsequently converted to LDH. The lactate is then taken up into the neuronal cytoplasm via MCT2, where it increases neuronal NADH and ATP production, thereby maintaining neuronal energy homeostasis when glucose uptake is compromised (Proia et al., 2016).

PAS staining results demonstrated that both apigenin and NE increased glycogen synthesis in astrocytes. This effect may be attributed to the activation of distinct signaling pathways: apigenin may act through GPR30 receptors on astrocytes to activate the PI3K-Akt-GSK3β signaling cascade, whereas NE may activate the cAMP/PKA/CREB pathway, both of which converge to enhance GS activity and promote glycogen synthesis (Bak et al., 2018). Notably, these findings reflect a dynamic balance between glycogen synthesis and breakdown rather than a static increase in glycogen stores, and glycogen turnover may better represent the metabolic state than content alone.

Targeted metabolomics results revealed that apigenin alone did not significantly promote lactate production in astrocytes, whereas NE treatment exhibited the opposite effect, suggesting that the apigenin-induced increase in hippocampal lactate production is likely mediated through its downstream enhancement of LC-NE signaling. Furthermore, inhibition of hexokinase with 2-DG reduced G-6-P and UDPG levels, whereas inhibition of glycogen phosphorylase with CP-316819 decreased all of the above metabolites. Targeted metabolomics results revealed that apigenin alone did not significantly promote lactate production in astrocytes, whereas NE treatment exhibited the opposite effect, suggesting that the apigenin-induced increase in hippocampal lactate production is likely mediated through its downstream enhancement of LC-NE signaling. Furthermore, inhibition of hexokinase with 2-DG reduced G-6-P and UDPG levels, whereas inhibition of glycogen phosphorylase with CP-316819 decreased all of the above metabolites. These findings indicate that apigenin promotes lactate production by enhancing LC-NE signaling, which in turn stimulates glycogenolysis in astrocytes. The resulting lactate is subsequently shuttled into neurons via the ANLS, where it is converted to pyruvate by LDH and ultimately enters the TCA cycle for aerobic metabolism to generate ATP required for neuronal activity (Calì et al., 2019).

It should be noted that only male mice were used in this study, in order to avoid the influence of hormonal fluctuations across the estrous cycle on behavioral and metabolic readouts, particularly given the phytoestrogenic nature of apigenin. Since AD is more prevalent in women, future studies incorporating both sexes, with careful control of the estrous cycle, are necessary to determine whether the neuroprotective effects of apigenin are sex-dependent.

Given that aging is the strongest risk factor for AD, longitudinal studies examining the effects of apigenin at different stages of aging are clearly warranted. We speculate that aging may attenuate the responsiveness of the GPR30/PLCβ1-Ca^2+^-Syt1 pathway, owing to age-related declines in estrogen receptor signaling, noradrenergic locus coeruleus integrity, and astrocytic metabolic capacity. Consequently, the efficacy of apigenin in aged animals may be reduced, and higher doses or longer treatment durations may be required. Whether apigenin exerts comparable effects in aged animals remains to be determined, and we have now stated this as an important direction for future research.

It should be emphasized that the D-galactose-induced model primarily recapitulates aging and aging-related cognitive decline rather than the full spectrum of AD pathology, as amyloid plaques and tau tangles were not evaluated in this study. Therefore, the present findings should be interpreted within the context of aging-related cognitive impairment, and further validation in authentic AD models is warranted.

## 5 Conclusion

Api improved learning and memory deficits in D-galactose-induced model mice by promoting hippocampal NE release and lactate production, thereby influencing multiple energy metabolism pathways, including the TCA cycle, alanine, aspartate and glutamate metabolism, and others, with efficacy comparable to that of G1. Further mechanistic investigations revealed that apigenin acts on the estrogen receptor GPR30 in noradrenergic neurons to regulate the PLCβ1-Ca^2+^-Syt1 signaling pathway, thereby promoting NE release. Moreover, the downstream effects of apigenin on the LC → HPC-NE axis were found to enhance astrocytic lactate levels by promoting glycogenolysis rather than glycolysis. Collectively, these results indicate that apigenin improves hippocampal energy metabolism through enhancement of the astrocyte–neuron lactate shuttle (ANLS), thereby contributing to its neuroprotective effects in D-galactose-induced cognitive impairment (Figure 6).

**FIGURE 6:**
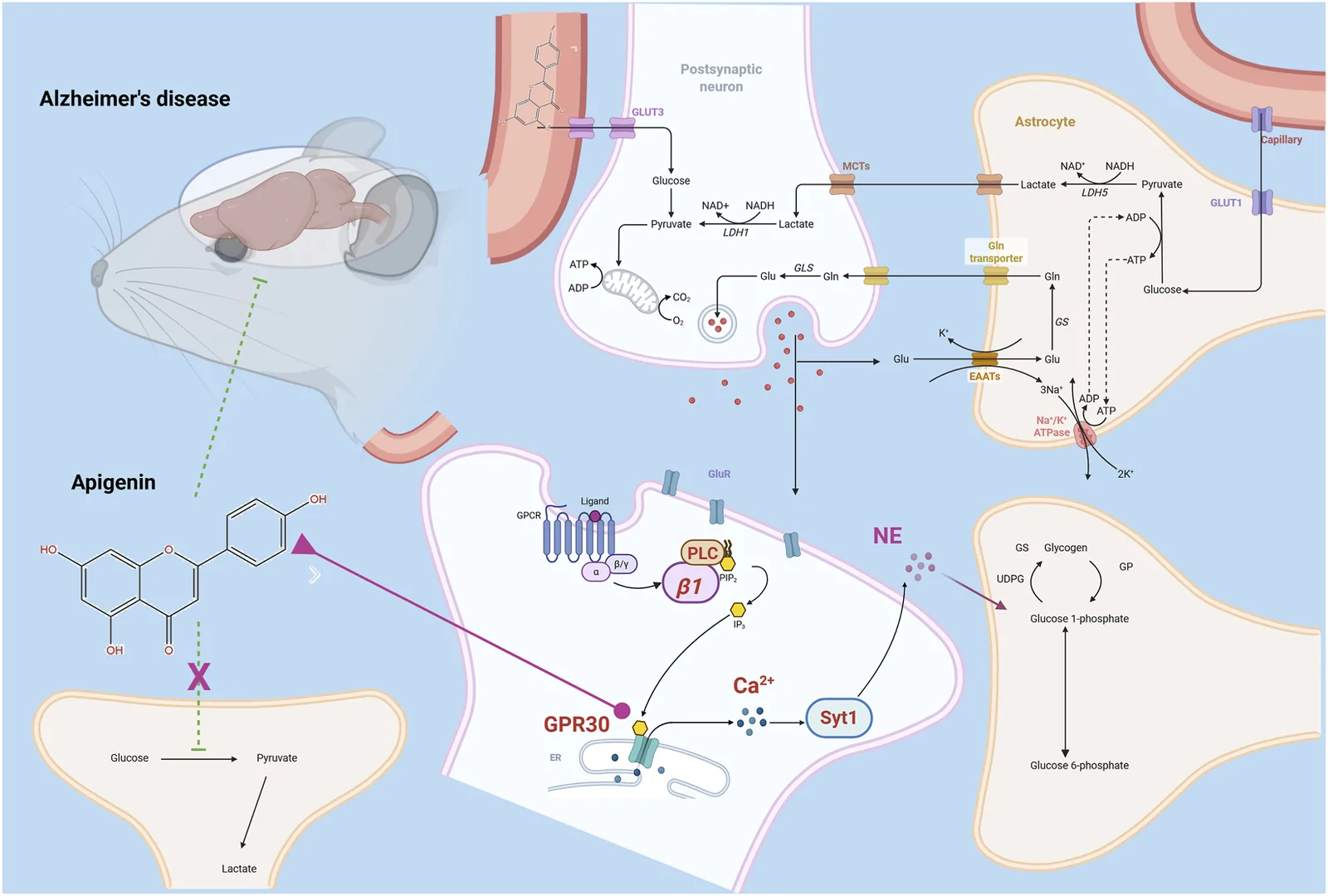
Apigenin promotes neuronal norepinephrine synthesis via GPR30/PLCβ1–Ca^2+^–Syt1 signaling, enhancing astrocytic lactate production and Conferring neuroprotection.

## Data Availability

The original contributions presented in the study are included in the article/supplementary material, further inquiries can be directed to the corresponding author.

## Glossary

AD: Alzheimer’s disease
ANLS: Astrocyte - Neuron Lactate Shuttle
ATP: Adenosine TriPhosphate
Aβ: Amyloid β-protein
CETSA: Cell Thermal Shift Assay
CNS: Central Nervous System
DA: Dopamine
DβH: Dopamine-β-hydroxylase
ER: Estrogen receptor
ERβ: Estrogen receptor β
ESI: Electro Spray Ionization
F-6-P: Fructose - 6 - Phosphate
G-1-P: Glucose - 1 - Phosphate
G-6-P: Glucose - 6 - Phosphate
GS: Glycogen Synthase
GSH: Glutathione
GP: Glycogen Phosphorylase
GPR30: G protein-coupled receptor 30
HPC: Hippocampus
LC: Locus coeruleus
LDH: Lactate Dehydrogenase
LTD: Long Term Depression
LTP: Long Term Potentiation
m6A: N6 - methyladenosine
NE: Norepinephrine
OPLS-DA: Orthogonal Partial Least Squares- Discriminant Analysis
PCA: Principal Component Analysis
PLC β1: Phospholipase C β1
RSD: Relative Standard Deviation
Syt1: Synaptotagmin
TH: Tyrosine Hydroxylase
UDPG: Uridine Diphosphate Glucose
UPLC-Q-TOFMS/MS: Ultra Performance Liquid Chromatography- Quadrupole-Time of Flight-Mass Spectrometer/Mass Spectrometer
UPLC-TQDMS/MS: Ultra Performance Liquid Chromatography- Triple Quadrupole-Mass Spectrometer/Mass Spectrometer
WB: Western blot
3-PG: 3 - Phosphoglycerate
